# Multimodal Sensors with Decoupled Sensing Mechanisms

**DOI:** 10.1002/advs.202202470

**Published:** 2022-07-14

**Authors:** Ruoxi Yang, Wanqing Zhang, Naveen Tiwari, Han Yan, Tiejun Li, Huanyu Cheng

**Affiliations:** ^1^ School of Mechanical Engineering Hebei University of Technology Tianjin 300401 P. R. China; ^2^ Department of Engineering Science and Mechanics The Pennsylvania State University University Park PA 16802 USA

**Keywords:** cross‐sensitivity, decoupling sensing mechanisms, multimodal sensors, multiple input signals

## Abstract

Highly sensitive and multimodal sensors have recently emerged for a wide range of applications, including epidermal electronics, robotics, health‐monitoring devices and human–machine interfaces. However, cross‐sensitivity prevents accurate measurements of the target input signals when a multiple of them are simultaneously present. Therefore, the selection of the multifunctional materials and the design of the sensor structures play a significant role in multimodal sensors with decoupled sensing mechanisms. Hence, this review article introduces varying methods to decouple different input signals for realizing truly multimodal sensors. Early efforts explore different outputs to distinguish the corresponding input signals applied to the sensor in sequence. Next, this study discusses the methods for the suppression of the interference, signal correction, and various decoupling strategies based on different outputs to simultaneously detect multiple inputs. The recent insights into the materials' properties, structure effects, and sensing mechanisms in recognition of different input signals are highlighted. The presence of the various decoupling methods also helps avoid the use of complicated signal processing steps and allows multimodal sensors with high accuracy for applications in bioelectronics, robotics, and human–machine interfaces. Finally, current challenges and potential opportunities are discussed in order to motivate future technological breakthroughs.

## Graphical abstract

This review article examines the evolution of multimodal sensors in detecting numerous input signals from discrimination and interference suppression to decoupling. The decoupled mechanisms exploit various materials, structures, and sensing principles. The current limitations and future opportunities also inspire the next‐generation flexible and stretchable sensors with optimized sensing performance to truly decouple complex input signals/stimuli for practical applications.

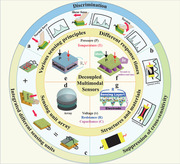

## 1 Introduction

Numerous sensory receptors underneath the skin (e.g., temperature receptors, pain receptors, and four types of mechanoreceptors^[^ ^1^ ^]^) can help humans perceive the external environment. Inspired by the functions of human skins, flexible electronic devices are designed with a monomodal function to detect force, temperature, humidity, among others. Due to their sensing performance to different input signals, these devices find applications in human–machine interfaces,^[^ ^2^ ^]^ robotics,^[^ ^3^ ^]^ prostheses,^[^ ^4^ ^]^ and healthcare devices.^[^ ^5^, ^6^ ^]^ To further advance flexible sensors to mimic the human skin that possesses a wide spectrum of mechanical properties and multiple sensing capabilities, multimodal flexible and stretchable sensors are proposed to measure multiple external stimuli by electrical signals (e.g., capacitance, resistance, current, or voltage). The multimodal sensing of the flexible sensors to multiple stimuli has been realized with active materials that respond to multiple physical stimuli or with delicate structures that show various deformations in sensing different mechanical stimuli. For example, graphene oxide (GO) or reduced graphene oxide (rGO) are very sensitive to humidity, chemicals and temperature due to their abundant surface functional groups, including hydroxyl, carboxyl and epoxy groups. On the other side, the force‐induced structures of the active layer in resistive sensors such as interlocking,^[^ ^7^, ^8^ ^]^ and helix^[^ ^9^, ^10^ ^]^ cause changes in conductive pathways and then resistances under mechanical stimuli. The development of multimode sensors is enabled by a variety of materials and structures, while signal recognition determines availability when numerous stimuli are delivered. As multimodal sensors are sensitive and respond to various input signals, it is vitally important to exploit them with decoupled sensing mechanisms for detecting the target signal without being affected by the cross‐sensitivity. Although signal processing sometimes can be helpful to minimize the effect of interference, multimodal sensors with decoupled sensing mechanisms can also reduce the complexity of the signal process.^[^ ^11^ ^,^ ^12^ ^]^

**Figure 1:**
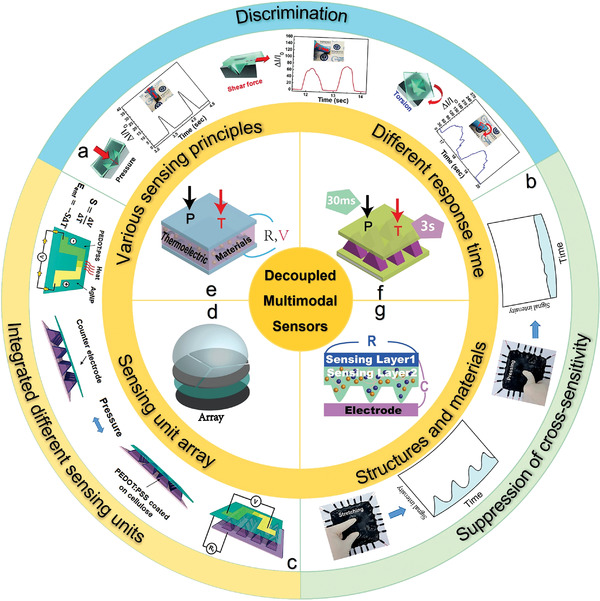
Schematic summarization of the development of multimodal sensors regarding decoupling mechanisms among different signals. a) Tactile sensor distinguishes different mechanical stimuli by signal patterns. Reproduced with permission.^[^^]^ Copyright 2018, Wiley‐VCH. b) Pressure‐insensitive strain sensor. Reproduced with permission.^[^ ^14^ ^]^ Copyright 2016, Nature Publishing Group. c) Integrated thermoelectric‐based temperature sensor and piezoresistive pressure sensor minimize interference with each other. Reproduced with permission.^[^ ^15^ ^]^ Copyright 2017, The Royal Society Chemistry. d) Polydimethylsiloxane (PDMS) bump‐based sensing array decouples 3D forces. e) Thermoelectric sensor to decouple pressure (P) and temperature (T) by different sensing principles. f) Multimodal sensor to decouple temperature (T) and pressure (P) by different response times. g) Bifunctional sensor based on a shared electrode to decouple different stimuli.

This review article examines the evolution of multimodal sensors in detecting numerous input signals (**Figure** **1**) from discrimination (Figure 1a) and interference suppression (Figure 1b) to decoupling (Figure 1c–g). The decoupled mechanisms exploit different sensing units (Figure 1c), array layout (Figure 1d) and single sensing unit enabled by materials with various principles (Figure 1e), different response times (Figure 1f) and combined structures and materials (Figure 1g). The current limitations and future opportunities will also be discussed to provide inspiration for the next‐generation flexible and stretchable sensors with optimized performance to truly decouple complex input signals/stimuli for applications in bioelectronics, robotics, and human–machine interfaces.

## 2 Discrimination Methods of Different Signals

Flexible sensors with active materials and delicate structures output varying electronic signals to different input stimuli. Apart from sequentially applying the input stimuli to avoid cross‐sensitivity^[^ ^9^, ^16^, ^17^, ^18^, ^19^, ^20^, ^21^, ^22^, ^23^ ^]^ the other efforts that include the use of different signal patterns^[^ ^7^, ^8^, ^13^, ^24^, ^25^ ^]^ opposite signal change direction,^[^ ^26^, ^27^, ^28^, ^29^, ^30^, ^31^, ^32^ ^]^ different electronic signal transducing types,^[^ ^33^, ^34^ ^]^ and the analysis of combined outputs.^[^ ^35^ ^]^

**Figure 2:**
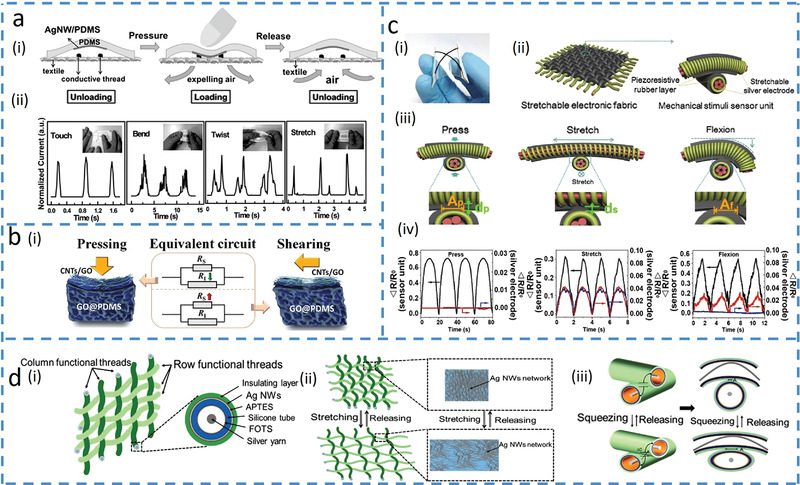
Asynchronous signal discrimination methods. a) Cloth‐based electronic skin (E‐skin) outputs different signal patterns under various forces. Reproduced with permission.^[^ ^24^ ^]^ Copyright 2016, Wiley‐VCH. i) Schematics of the cloth‐based sensor under pressure. ii) Changes in the relative current waveforms with various mechanical stimuli. b) Normal‐tangential force sensor with opposite resistance response. Reproduced with permission.^[^ ^27^ ^]^ Copyright 2018, Wiley‐VCH. i) Schematics of the sensor deformations upon normal pressure and lateral shear force. ii) Equivalent circuits, where *R*_S_ represents the resistance of the surface sublayer‐carbon nanotubes (CNTs)/graphene oxide (GO), and *R*_I_ represents the resistance of the inner sublayer‐GO/PDMS. c) Electronic fabric artificial skin that measures pressure, strain, and flexion by a combined analysis of different output signals from multiple electrodes. Reproduced with permission.^[^ ^35^ ^]^ Copyright 2015, Wiley‐VCH. i) Optical image of a sensor unit on a polyethylene terephthalate substrate. ii) Illustration of fibrous E‐skin and a single sensing unit. iii) The deformation at the contact point of the sensor unit with applied pressure, stretching, and flexion. *A*_p_ and *d*_p_ are the contact area and thickness with applied pressure, *d*_s_ is the thickness under stretching, and *A*_f_ is the contact area under flexion. iv) Changes in the relative resistances of the three electrodes over time for repeated pressure, strain, and flexion loadings. d) Multifunctional woven tactile sensor array. Reproduced under the terms of the Creative Commons CC BY License.^[^ ^34^ ^]^ Copyright 2017, The Authors, published by MDPI. i) Structural demonstration of the multifunctional woven sensor. ii) Schematic of the inner silver layer at resistant sensing mode for measuring strain. iii) Schematic illustration of the capacitor for pressure detection.

Early efforts explore different output signal patterns from multimodal tactile sensors for the identification of different mechanical stimuli (e.g., pressure, bending, strain, and torsion). For example, a resistive electronic skin (E‐skin) outputs various current patterns to reveal the different mechanical stimuli by employing force‐dependent contact spots between conductive materials and electrodes.^[^ ^24^ ^]^ With two bottom electrodes fixed on the rough textile, a PDMS layer with silver nanowires (Ag NWs) embedded on the surface serves as a conductive film suspended above (**Figure** **2**a‐i). Applied mechanical stimuli induce different contact spots between the Ag NWs network and two electrodes, leading to various current responses (Figure 2a‐ii). The opposite changes in the direction of output electrical signals can also be used to distinguish two different input stimuli. For example, a normal‐tangential force sensor based on carbon nanotubes (CNTs)/GO@PDMS is developed for outputting direction‐dependent signals.^[^ ^27^ ^]^ The sensor is designed with a two‐sublayered conductive architecture, consisting of a top layer (CNTs/GO) and a bottom porous layer (GO@PDMS), with the two resistors (*R*_s_ and *R*_I_) in parallel connection (Figure 2b‐i,ii). The pressure allows the GO sheets in the GO/PDMS porous structure to approach and overlap to decrease *R*_I_, whereas the tangential shear force causes an expansion of the microcracks between GO sheets and sliding of CNTs across each other with bonded GO sheets to increase *R*_s_. Compared to the single output, accuracy can be improved when the sensor detects stimuli with multiple sensing units. A fabric E‐skin employs the simultaneously measured resistance signal from three units to distinguish pressure, lateral strain, and flexion (Figure 2c‐i).^[^ ^35^ ^]^ The individual sensing units or the sensor electrodes are formed by coating Ag NWs with conductive rubber, followed by cross‐contacting two sensor electrodes (Figure 2c‐ii). The distinct contact areas between the electrode and conductive rubber as well as the shape deformation of the sensor electrodes from different mechanical stimuli (Figure 2c‐iii) result in different resistance responses from three sensing units (Figure 2c‐iv). Furthermore, the multimodal sensor can transduce different stimuli into different electric signals, such as a woven textile‐based device consisting of a resistive sensor and a capacitive sensor to detect tensile strain and pressure (Figure 2d‐i).^[^ ^34^ ^]^ The Ag NWs coated tube provides the resistor (electrode) to detect tensile strain as it changes the connection between Ag NWs (Figure 2d‐ii), whereas two perpendicular resistors with an insulating elastomer layer form a capacitor to detect pressure as it changes the overlapping area (Figure 2d‐iii).

Although the aforementioned methods can effectively distinguish input stimuli when applied in sequence, they are not applicable for simultaneously applied input stimuli as they may interact or even directly cancel with each other. Efforts to minimize cross‐sensitivity and further decouple simultaneously applied input signals have led to the development and exploration of novel materials, structures, sensing mechanisms, and sensor layouts in the truly decoupled multimodal sensors. The decoupled different input signals from the multimodal devices allow it to mimic natural skins for sensing complex environmental changes.

## 3 The Suppression of the Interference

The most straightforward method to accurately measure one input signal from multiple simultaneously applied stimuli is to suppress the influence of other input stimuli on the output signal(s). Because the flexible electronic device is highly elastic and deformable, different deformations such as stretching, bending, and compressing may contribute to the changes in electrical output signals. When the pressure is of concern, three important methods have been exploited to decouple it from other deformation modes for improved accuracy and specificity. These methods rely on the concentration control of active materials in the composite,^[^ ^14^, ^36^ ^]^ adjusting the morphology/structure modulation of the active/sensing materials,^[^ ^37^, ^38^ ^]^ and exploring the novel sensing mechanism.^[^ ^39^ ^]^

**Figure 3:**
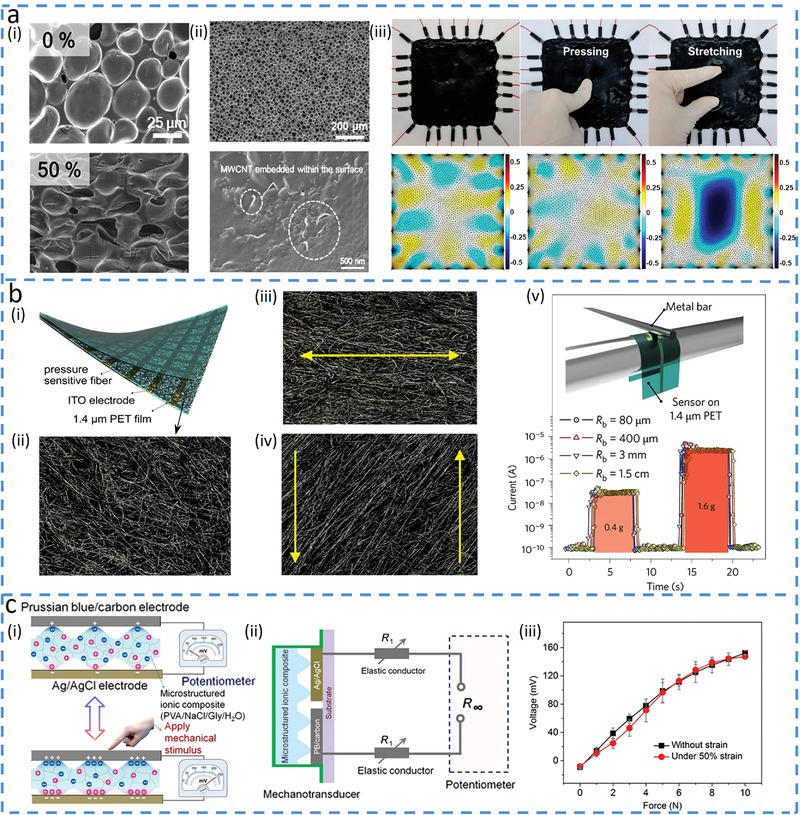
Decoupling target input by suppressing the influence of the other input signals. a) Pressure‐insensitive strain (PIS) sensor based on multiwalled carbon nanotubes (MWCNTs) and PDMS composite materials. Reproduced with permission.^[^ ^14^ ^]^ Copyright 2018, American Chemical Society. i) Scanning electron microscopy (SEM) images of the PIS sensor without (top) and with (bottom) tensile strain. ii) SEM images of the composite materials with a porous structure (top), and the zoomed version shows that most of the MWCNTs are embedded in the PDMS (bottom). iii) Photograph (top) and corresponding impedance analysis (bottom) of the PIS sensor under initial, pressured, and locally strained conditions. b) Transparent pressure sensor that is insensitive to bending. Reproduced with permission.^[^ ^37^ ^]^ Copyright 2016, Nature Publishing Group. i) Schematic diagram of the bending‐insensitive pressure sensor. ii) SEM image of the fiber layer. iii) SEM image of the fiber layer under tensile strain. iv) SEM image of the fiber layer under shear force. v) Sensor responds to pressure (0.4 and 1.6 g) at different bending radii (from 1.5 cm to 80 µm). c) E‐skin based on potentiometric mechanotransduction mechanism^[^ ^39^ ^]^ and its schematic illustration shown in (i). Two electrodes with reversible oxidation–reduction reactions are used to create a potential difference, where the microstructured ionic composite between electrodes alters the potential under an applied force. ii) Circuit model of the strain‐insensitive potentiometric sensor. iii) Almost invariable force sensing performance with or without 50% strain. Reproduced under the terms of the Creative Commons CC BY NC License.^[^ ^39^ ^]^ Copyright 2020, The Authors, published by The American Association for the Advancement of Science.

Composite materials are often leveraged as an active layer to sense deformation because the deformation‐induced changes in the percolation network of the composite give changes in the electrical outputs of the sensor. The concentration of active materials in the composite can be controlled to be below the percolation threshold to fabricate pressure‐insensitive sensors. For example, a strain sensor with a 0.7 wt% concentration of multiwalled CNTs (MWCNTs) in PDMS/MWCNTs composite shows reduced percolation pathways in the MWCNTs network and increased resistance as the strain increases (**Figure** **3**a‐i), but it shows a negligible change in resistance under pressure.^[^ ^14^ ^]^ The pressure‐insensitivity of the porous piezoresistive structure directly results from the low concentration of the MWCNTs, as most of the fillers embedded within PDMS can hardly form new conductive pathways even upon pressure (Figure 3a‐ii,iii). Interestingly, the composite with a high concentration of fillers can be used to fabricate bending‐insensitive sensors. Although bending often shifts the percolation and varies conductivity in a composite (due to reduced effective length),^[^ ^40^ ^]^ employing a high concentration of graphene in PDMS (above percolation threshold) results in a hollow structure‐based pressure sensor to avoid the shift of percolation in a composite under bending.^[^ ^36^ ^]^ Besides innovation in composite materials, the morphology of the active materials or structures of the sensing layer can be tailored to address bending interference. For example, nanofibers based on CNTs/graphene composite (Figure 3b‐i,ii) could adjust their alignment to accommodate applied bending deformations (Figure 3b‐iii,iv) and maintain their initial conductivity.^[^ ^37^ ^]^ The reduced strain in individual fibers from bending provides the piezoresistive pressure sensor with bending‐insensitive performance (Figure 3b‐v). In another example, a piezoresistive pressure sensor based on a porous elastomer sponge coated with CNT changes its overall arrangement of the microporous structure upon bending deformation, leading to negligible deformation in individual micropores to cause unnoticed changes of conductive pathways.^[^ ^38^ ^]^ The simple and low‐cost piezoresistive sensor to detect mechanical stimuli^[^ ^41^ ^]^ is also easily influenced by different strains, leading to the exploration of an open‐circuit to mitigate the strain interference.^[^ ^39^ ^]^ Between two active electrodes with reversible oxidation–reduction reactions to generate a potential difference, a microstructure electrolyte can increase its contact area with electrodes to decrease interfacial impedance under increased applied pressure (Figure 3c‐i). The potential difference changes generated from charge transfer can be obtained by a large‐value resistor (*R*) as the voltage across *R* measures the open‐circuit potential (Figure 3c‐ii). As the resistance of the elastic conductors or electrodes (*R*_1_) is much smaller than that of *R*, its changes during stretching deformation do not alter the measured open‐circuit voltage, leading to strain‐insensitive pressure measurements (Figure 3c‐iii).

## 4 Integrating Different Sensing Units for Decoupled Multimodality

**Figure 4:**
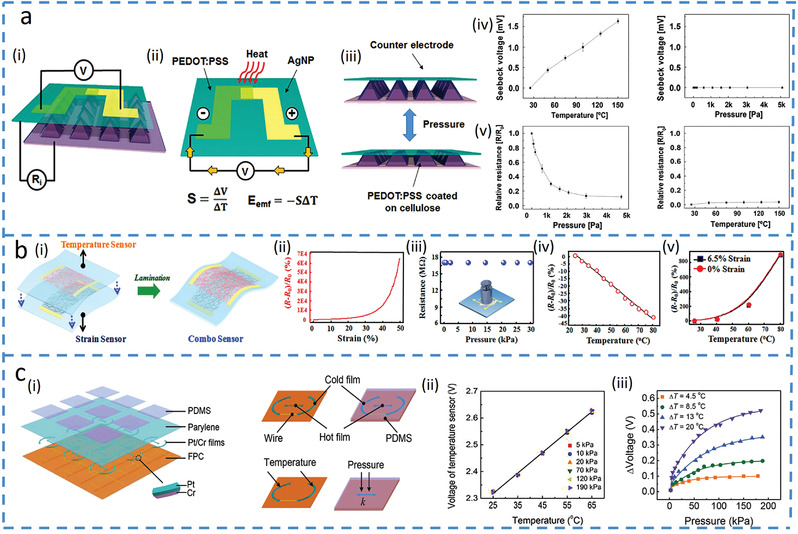
Integrated platforms with multiple sensors. a) Laminated nano‐cellulose tactile sensor. Reproduced with permission.^[^ ^15^ ^]^ Copyright 2017, The Royal Society Chemistry. i) Structural model with temperature (ii) and pressure (iii) sensing mechanisms. Responses from iv) temperature and v) pressure sensors to temperature and pressure. b) Silk‐derived E‐skin combined with pressure and temperature sensors. Reproduced with permission.^[^ ^42^ ^]^ Copyright 2017, American Chemical Society. i) Schematic showing an integrated temperature and pressure sensors (fabricated by intact continuous nanofiber structure and fractured nanofiber, respectively). ii) Relative resistance changes of the strain sensor versus strain. iii) Relative resistance changes of the temperature sensor versus pressure (not strain). Relative resistance changes of the iv) temperature and v) strain sensors versus temperature. c) Multifunctional E‐skin based on thermosensitive platinum. Reproduced with permission.^[^ ^43^ ^]^ Copyright 2017, Wiley‐VCH. i) Device structure of the E‐skin with the element descriptions. ii) Temperature response of the temperature sensor under different pressure. iii) Pressure response of the pressure sensor under different temperatures.

The most commonly used approach for simultaneously detecting distinct input signals is to integrate multiple sensing units in‐plane (i.e., tiling)^[^ ^43^, ^44^, ^45^, ^46^ ^]^ or out‐of‐plane (i.e., laminating),^[^ ^15^, ^42^, ^44^, ^47^, ^48^, ^49^, ^50^, ^51^, ^52^, ^53^, ^54^, ^55^, ^56^, ^57^, ^58^, ^59^ ^]^ into a sensing platform, in which each sensor targets for individual sensing functions. For instance, individual sensors with thermoelectric or thermal‐resistant effects are used to measure temperature input. Besides, those with triboelectric, piezoresistance, or piezoelectric effects are employed to respond to the pressure. The integration of these sensors with different sensing principles has resulted in multi‐sensing platforms, including the combination of thermal resistive and piezoelectric,^[^ ^59^ ^]^ piezoresistive and thermoelectric,^[^ ^15^, ^50^ ^]^ piezoelectric and thermoelectric,^[^ ^57^ ^]^ and piezoelectric and piezoresistive sensors.^[^ ^58^ ^]^ For example, a multimodal tactile sensor with temperature and pressure sensors based on different operation principles can simultaneously detect the two with minimized interference (**Figure** **4**a‐i).^[^ ^15^ ^]^ Temperature sensor based on poly(3,4‐ethylenedioxythiophene)‐poly(styrenesulfonate) (PEDOT:PSS) and silver nanoparticles (Ag NPs) composite generates a thermoelectric voltage in the thermocouple (Figure 4a‐ii), whereas the pressure sensor with micro‐sized pyramids changes its resistance due to pressure‐induced changes in contact area (Figure 4a‐iii). The different sensing mechanisms result in no interference even when temperature and pressure are simultaneously applied to the sensing platform (Figures 4a‐iv,v). Besides exploiting different materials for varying sensing mechanisms, the same material can also be patterned into distinct structures to provide different sensing mechanisms with significantly minimized interference,^[^ ^46^, ^53^, ^54^, ^55^, ^60^ ^]^ For example, the CNT/PDMS composite with a wrinkled structure on top of a sponge structure can selectively detect humidity and pressure, respectively.^[^ ^60^ ^]^ The humidity sensor based on a wrinkled structure with the treatment of ultraviolet and ozone (UVO) to improve the hydrophilic and surface wetting area responds to increased humidity by enlarging the polymer chain matrix and the reduced connection of CNTs shows decreased conductivity. Meanwhile, the composite with porous structures in the bottom layer provides a piezoresistive sensor to detect pressure, which is almost insensitive to humidity. The humidity sensor is not nearly affected by pressure either as the pressure transmits through the top layer to induce changes in the bottom porous sensing layer.

Different monomodal sensors with the same material in the multi‐sensing platform may experience cross‐sensitivity, but it is still possible to decouple if cross‐sensitivity only appears in one of the two monomodal sensors, providing a way to solve for both.^[^ ^42^, ^51^ ^]^ For example, silk‐nanofiber‐derived carbon in continuous and fractured nanofiber membrane forms is used as active materials to detect temperature and strain, respectively (Figure 4b‐i).^[^ ^42^ ^]^ The increased strain decreases the contact points between fractured forms of the silk‐nanofibers to increase the resistance in the strain sensor (Figure 4b‐ii), but it is claimed to have no influence on the temperature sensor (though the presented result is pressure) (Figure 4b‐iii). Meanwhile, elevated temperatures increase carrier hopping and tunneling conduction to increase the conductance of nanofiber, leading to reduced resistance in the temperature sensors (Figure 4b‐iv). Therefore, the temperature can be first determined from the temperature sensor. Though the temperature also affects the strain sensor because of the thermal expansion of the PDMS substrate to change its absolute value, this value can be obtained at the determined temperature (Figure 4b‐v), which can then be used to yield the strain from the calibration curve of the strain sensor at the given temperature (Figure 4b‐ii). An alternative method to eliminate interference is using a reference sensor that is only sensitive to one input signal,^[^ ^43^, ^61^, ^62^ ^]^ For example, a platinum (Pt)‐based multifunctional E‐skin based on thermosensation under an applied voltage can accurately detect pressure with the assistance of a pressure‐insensitive temperature sensor (Figure 4c).^[^ ^43^ ^]^ The applied voltage on Pt below the porous elastomer in the pressure sensor produces high electrical power to heat up the elastomer that deforms under the applied pressure, which increases its thermal conductivity to lower the temperature of the Pt (Figure 4c‐i). The resistance of the Pt pressure sensor decreases with the reduced temperature, which can be measured by a voltage signal and compared with the initial voltage without pressure to calculate the applied pressure. In contrast, configuring the Pt to a high‐resistance temperature sensor provides low Joule heating under the applied voltage and the resistance change of the Pt is only sensitive to ambient temperature (Figure 4c‐ii). Although the ambient temperature also affects the pressure sensor through heat transfer (Figure 4c‐iii), the temperature measured directly by the temperature sensor can be used to correct the outputs from the pressure sensor. It is worth noting that heterogeneous sensing units with varied architectures, materials, and mechanisms in the multi‐sensing platform are often associated with high fabrication costs and present challenges for the applications of multimodal sensors.

## 5 Array Layout for Decoupled Multimodality

**Figure 5:**
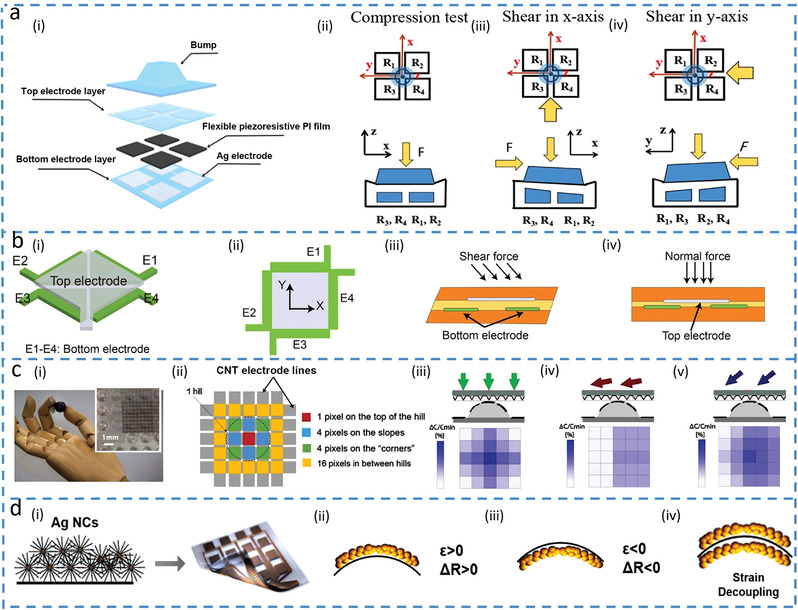
Array layout for decoupled multimodality. a) Flexible sensor based on CNT/PDMS composites to measure 3D force. Reproduced with permission.^[^ ^65^ ^]^ Copyright 2018, Springer Nature. i) Sensor in an exploded view and its demonstration to measure ii) pressure alone or iii,iv) shear forces in the *x*‐ or *y*‐axis with the pressure. b) Flexible capacitive sensor to measure pressure and shear forces.^[^ ^68^ ^]^ i) Schematic diagram, ii) layout, and its sensing principles for detecting shear (iii) and normal forces (iv). c) Bioinspired capacitive E‐skin to monitor the direction of force. Reproduced with permission.^[^ ^69^ ^]^ Copyright 2018, The American Association for the Advancement of Science. i) Optical image and ii) sensor placement of the E‐skin. Color maps show the real‐time discrimination of iii) normal force only, iv) shear force only, and v) combined normal and shear forces. d) Mirror‐stacked layer designed for decoupling bending from other stimuli such as temperature. Reproduced with permission.^[^ ^70^ ^]^ Copyright 2019, American Chemical Society. i) Schematic diagram of the sensor with ii) increased or iii) decreased length (and resistance) in the upper or lower part for iv) canceling the effect of bending.

Positing the identical sensing units in an array at different locations on an elastomer (round mesa^[^ ^63^ ^]^ and cubed PDMS bump^[^ ^64^, ^65^, ^66^ ^]^) also provides a platform to decouple 3D force/pressure signals. For example, the array of four carbon black resistive sensors on polyimide (PI) integrated with a PDMS bump on the top (**Figure** **5**a‐i) can detect both normal and shear forces.^[^ ^65^ ^]^ While the same output from four units indicates the same normal pressure (Figure 5a‐ii), the combined pressure and shear along the positive *x*‐ (or *y*‐) direction result in a tilted bump to induce compressive/tensile stress in the sensing units positioned at the positive/negative *x*‐ (or *y*‐) direction (Figure 5a‐iii,iv). The output difference between two units in the shear direction can be used to calculate the shear force. The sensing units themselves can also be fabricated into 3D shapes to avoid the use of the additional bump layer. For example, the pre‐stretch and releases strategy can form a stereostructure of the PI film with a Si nanomembranes‐based sensor bonded to an elastomeric substrate.^[^ ^67^ ^]^ Without using a bump layer, the soft capacitive sensor consisting of four capacitors with the individual bottom electrodes and a common square‐shaped top electrode can also be employed to decouple pressure and shear force (Figure 5b‐i,ii).^[^ ^68^ ^]^ The applied shear force along the positive *x*‐direction changes the capacitance of two by an equal and opposite value while leaving the other two unchanged, whereas the pressure reduces the gap between the top and bottom electrodes to give the same capacitance change in all four capacitors (Figure 5b‐iii,iv).

Increasing the number of the sensing units in the array can potentially provide more detailed information (direction, distribution, or contact position),^[^ ^69^, ^71^, ^72^ ^]^ for delicate manipulation activities (e.g., multi‐fingered hands with several degrees of freedom). The biomimetic E‐skin (Figure 5c‐i) with a dielectric layer between a bottom electrode on a 3D hill structure and 25 pyramid‐based top electrodes (Figure 5c‐ii) can output a varied response in each capacitor depending on its location upon the anisotropic deformation (from combined normal and shear forces).^[^ ^69^ ^]^ Hence, the output map from the array reflects the distribution of the applied force (Figure 5c, iii–v).

Mirror‐stacking two identical sensing units on both sides of a thin film provides a simple method to decouple bending strain from other stimuli.^[^ ^70^, ^73^, ^74^ ^]^ Although the Ag nanocrystal is sensitive to both temperature and bending strain, mirror‐stacking two sensing units in bifunctional sensors results in positive/negative strain in the upper/lower layer upon bending for canceling the bending effect to measure the temperature (Figure 5d).^[^ ^70^ ^]^ The difference between the two sensing layers gives the bending strain. Similarly, mirror‐stacking two liquid metal‐based sensors in microchannels exhibit the same (or opposite) signal variations for uniaxial (or bending) strain, decoupling the tensile and bending strain.^[^ ^73^ ^]^ Moreover, laminating two identical sensing units with one positioned along the longitudinal and the other along the transverse direction can decouple strains from different directions. Ag NWs sensor with percolation networks on two corrugated substrates created by the pre‐strain strategy can independently detect the strain along these two directions.^[^ ^54^ ^]^ When the applied strain is smaller than the pre‐strain along the pre‐strain direction, the sensor positioned along this direction exhibits no resistance change, but the one along the other direction experiences an increased resistance because of the increased distance between nanowires.

## 6 Novel Materials with Multiple Sensing Mechanisms in a Single Sensing Unit

**Figure 6:**
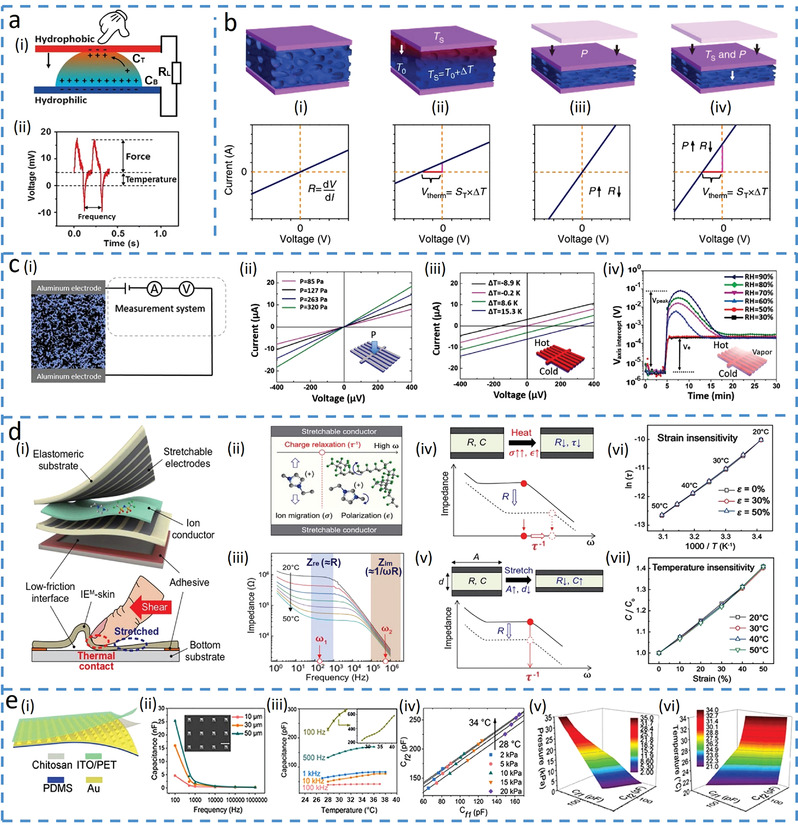
Novel materials with multiple sensing principles in a single unit. a) Dual‐modal sensor built by a liquid droplet. Reproduced with permission.^[^ ^75^ ^]^ Copyright 2016, Wiley‐VCH. i) Sensing principle of the droplet sensor. ii) Variation of voltage over time when detecting temperature and pressure simultaneously. b) Dual‐parameter sensor based on thermoelectric materials^[^ ^76^ ^]^ at i) initial condition and after ii) temperature loading (with changed intercept) and iii) pressure loading (with shifted slope). iv) The sensor detects both the temperature and pressure with variations from the intercept and slope of the current‐voltage (I–V) curve. Reproduced under the terms of the Creative Commons CC BY License.^[^ ^76^ ^]^ Copyright 2015, The Authors, published by Springer Nature. c) Multiparameter sensor based on ionic aerogels. Reproduced under the terms of the Creative Commons CC BY License.^[^ ^77^ ^]^ Copyright 2019, The Authors, published by Wiley‐VCH. i) Schematic illustration of the pressure‐temperature‐humidity sensor with I–V curves exhibiting ii) different slopes for varying pressures and iii) different voltage axis intercepts for various temperatures. iv) Voltage as a function of time for different humidity values (Δ*T* = 10*k* added at 4 min). d) Artificial multimodal receptors that can differentiate strain and temperature. Reproduced with permission.^[^ ^12^ ^]^ Copyright 2020, The American Association for the Advancement of Science. i) Schematic illustration and ii) frequency‐dependent behavior of the ion conductor in different electric fields. iii) Bode plots for an ion conductor with a 5 wt% ion concentration at different temperatures. iv) Demonstration of the Bode plot with an increased (cut‐off) charge relaxation frequency (*τ* ^−1^) and decreased resistance after applying temperature. v) Bode plot displaying a parallel downshift when the device is stimulated by tensile strain. vi) Changes in the charge relaxation time (ln (*τ*)) as a function of *T* ^−1^ (*T*, temperature). vii) Relationship between the relative changes in capacitance and the tensile strain at various temperatures. e) Frequency‐enabled decouplable dual‐modal sensor. i) Schematic of the bimodal sensor based on chitosan. Reproduced with permission.^[^ ^78^ ^]^ Copyright 2020, IEEE. ii) Capacitance measurement of the sensor with different‐sized micropyramids under different frequencies at 80 kPa. iii) Temperature sensing performance varied under different frequencies. iv) Capacitance changes at 1 kHz (*C*_f1_) and 500 Hz (*C*_f2_) with both temperature and pressure stimuli. 3D map produced by linear interpolation to estimate pressure (v) and temperature (vi).

To reduce the complexity and manufacturing costs of using heterogeneous sensing materials and devices in a multi‐sensing platform, it is of high interest to exploit novel materials with multiple sensing mechanisms in a single sensing unit. For instance, K_3_[Fe(CN)_6_]/K_4_[Fe(CN)_6_] solution with thermogalvanic property^[^ ^79^ ^]^ can be used for temperature sensing and its combination with two conducting plates can be further used to measure force due to reverse electrowetting^[^ ^80^ ^]^ under pressure. An electric double‐layer (EDL) capacitor is formed by sandwiching the liquid droplet between a bottom hydrophilic plate and a top hydrophobic plate, with the former to fix the droplet location and the latter to maintain the droplet shape after deformation (**Figure** **6**a‐i).^[^ ^75^ ^]^ Temperature sensing induced by the thermogalvanic property of the droplet allows the device to directly generate a voltage change. On the other hand, the force‐sensing results from the alternating pulse voltage induced by the pressure that modulates the droplet shape and induces reverse electrowetting, which leads to the charging and discharging processes of the capacitor to generate a pulse voltage (Figure 6a‐ii). Thus, the direct and pulse voltage readouts can simultaneously measure the temperature and pressure.

Thermoelectric materials combined with a porous elastomer can be used as the responsive component to simultaneously measure force and temperature based on piezoresistive and thermoelectric effects.^[^ ^56^, ^76^, ^81^ ^]^ In particular, the resistance as the slope and the voltage as the shift in the single current–voltage (*I*–*V*) curve provide two independent outputs to decouple two input signals. For example, immersing PEDOT:PSS into a porous polyurethane microstructure frame results in a dual‐modal sensor to detect temperature and pressure (Figure 6b).^[^ ^76^ ^]^ Compared with the initial state of the sensor (Figure 6b‐i), the *I*–*V* curve shifts upwards for an increased temperature according to the thermoelectric effect (Figure 6b‐ii). The applied pressure bends the microstructured frame and changes the resistance of the active layer, leading to changes in the slope of the *I*–*V* curve (Figure 6b‐iii). For the simultaneously applied temperature and pressure stimuli, the voltage and resistance from the shift and slope of the *I*–*V* curve immediately provide the temperature and pressure values (Figure 6b‐iv). This mechanism also allows the thermoelectric titanium carbide (MXene)‐Ag NW‐PEDOT:PSS‐tellurium nanowire (Te NWs) composite on a polyurethane substrate to decouple strain and temperature.^[^ ^82^ ^]^ The Te NWs/PEDOT:PSS hybrid is used to detect the temperature from strain‐insensitive voltage, whereas the conductive MXene‐Ag NW network is employed to sense the strain that induces the temperature‐insensitive resistance change due to crack‐propagation without interference. Incorporating PEDOT:PSS in an ion‐electron conducting porous aerogel can leverage its electronic and ionic Seebeck effect^[^ ^83^ ^]^ to detect humidity, which results in a sensor to decouple pressure, temperature and humidity (Figure 6c).^[^ ^77^ ^]^ The electronic and ionic Seebeck effect of PEDOT:PSS allows the temperature‐induced thermoelectric voltage to first increase to a peak due to ion thermo‐diffusion in the humid environment, followed by a decrease to stabilize at a value that solely depends on the temperature, decoupling humidity from temperature. When the pressure, temperature and humidity are simultaneously applied, the pressure and temperature can be obtained from the resistance (Figure 6c‐ii) and voltage (Figure 6c‐iii) shift of the steady‐state *I*–*V* curve, whereas the peak in the voltage versus time plot gives the humidity value (Figure 6c‐iv).

Ionic or proton‐based materials with frequency‐dependent characteristics under an applied alternative current,^[^ ^84^, ^85^ ^]^ can also be used for multimodal sensing.^[^ ^12^, ^78^ ^]^ For instance, 1‐ethyl‐3‐methylimidazoliumbis(trifluoromethylsulfonyl)imide (EMIM TFSI) ionic conductor combined with two stretchable electrodes in a multimodal receptor decouples strain and temperature (Figure 6d‐i).^[^ ^12^ ^]^ As the migration and polarization of ions take place at low and high frequencies, respectively (Figure 6d‐ii), bulk resistance (*R* = *d*/*σA*) and capacitance (*C* = *εA*/*d*) exhibit different behaviors at different frequencies (Figure 6d‐iii) (*A*, *d*, *σ*, and *ε* for the area, thickness, ionic conductivity, and dielectric constant). The charge relaxation time (*τ* = *ε*/*σ* = *RC*)^[^ ^86^ ^]^ only depends on the altered ionic conductivity and dielectric constant (Figure 6d‐iv) is not affected by stretching because of the canceled dimensional parameter (Figure 6d‐v). Thus, *τ* can be utilized as a strain‐insensitive measurement of temperature based on Arrhenius behavior (Figure 6d‐vi). Although the capacitance is affected by both the temperature (via dielectric constant change) and strain (via shrinking), the normalized capacitance by a reference capacitance at the detected temperature becomes temperature‐insensitive (same rate of change at different temperatures) for detecting strain (Figure 6d‐vii). Chitosan film with proton conductivity also exhibits frequency‐dependency, which can be used to decouple pressure and temperature.^[^ ^78^ ^]^ With a chitosan‐based dielectric layer sandwiched between a top ITO electrode and a bottom Au‐coated PDMS micropyramid electrode, an EDL forms at the micropyramid/chitosan interface (Figure 6e‐i). The pressure‐induced deformation of the micropyramid changes the interfacial contact area to give a linear capacitance response. Meanwhile, the increased temperature increases the number and mobility of free ions to also give a linear increase of capacitance. However, the rotation speed of the proton becomes too slow to accumulate to the interface in the high‐frequency electric field, which leads to a decreased capacitance^[^ ^87^, ^88^ ^]^ (Figure 6e‐ii,iii). Therefore, the pressure and temperature‐induced capacitance can be measured at different frequencies (Figure 6e‐iv). By using linear interpolation, all the *C*_f1_ and *C*_f2_ can be obtained corresponding to the different pressures and temperatures (Figure 6e‐v,vi). The group data from *C*_f1_ and *C*_f2_ can be then used to determine the applied temperature and pressure.

**Figure 7:**
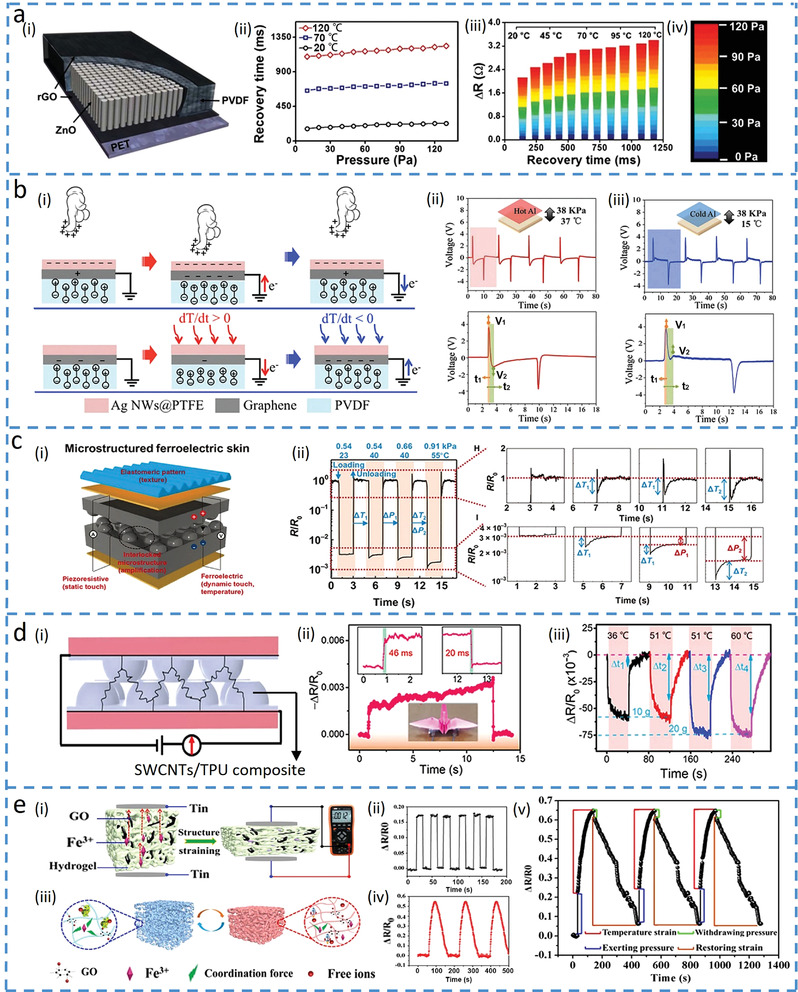
Materials with different response times to decouple temperature and force. a) Multifunctional tactile sensor based on ZnO/polyvinylidene fluoride (PVDF) film for detecting pressure and temperature. Reproduced under the terms of the Creative Commons CC BY License.^[^ ^89^ ^]^ Copyright 2015, The Authors, published by Springer Nature. i) Schematic of the sensor and ii) its recovery time changing with the applied pressure at different temperatures. iii) Color mapping to show the resistance changes as a function of recovery time, with iv) corresponding pressures applied to the sensor. b) Multi‐effect flexible tactile sensor in decoupling temperature and pressure. Reproduced with permission.^[^ ^90^ ^]^ Copyright 2019, Elsevier Ltd. i) Schematic of the tactile sensor for sensing pressure (top) and temperature (bottom). Voltage changes in the sensor when the pressure of 38 kPa is applied with a temperature of ii) 37 or iii) 15 °C. The enlarged view shows *V*_1_ generated by the temperature and pressure and *V*_2_ by the temperature only. c) Ferroelectric skins for detecting static/dynamic pressure and temperature. Reproduced under the terms of the Creative Commons CC BY NC License.^[^ ^91^ ^]^ Copyright 2015, The Authors, published by The American Association for the Advancement of Science. i) Schematic diagram of the ferroelectric skin and ii) its response (relative resistance changes) versus time for loading/unloading at various temperatures and pressures. d) Multifunctional E‐skin based on an interlocked quasi‐hemispherical micropattern array. Reproduced with permission.^[^ ^92^ ^]^ Copyright 2020 Elsevier B.V. ii) Response and recovery times with an object placed on top. iii) Change in relative resistance when the sensor is stimulated by both temperature and pressure. e) Dual‐mode sensor fabricated by an ionic hydrogel. Reproduced with permission.^[^ ^93^ ^]^ Copyright 2019, American Chemical Society. i) Pressure sensing mechanism and ii) its relative resistance changes upon pressure loading. iii) Sensing mechanism for temperature and iv) its change in relative resistance at 40 °C. v) Variation of relative resistance versus time upon both temperature and force loadings.

Besides active materials with multiple sensing mechanisms, the material with different response/recovery times may also be leveraged to decouple input stimuli such as temperature and pressure. For example, polyvinylidene fluoride (PVDF) with inherent pyroelectric and piezoelectric effects that depend on the polarization and the dipole moment can be used for sensing temperature and pressure in bimodal sensors. The temperature‐induced movement of the dipoles is slower^[^ ^94^ ^]^ to give increased response/recovery times, compared to that from the applied pressure, which can be leveraged to separate temperature and pressure. Furthermore, the addition of zinc oxide (ZnO) increased permittivity can enhance the pyroelectric and piezoelectric properties of PVDF due to increased polarization and dipole moment. The bimodal pressure and temperature sensor based on PVDF and ZnO nanorods with graphene electrodes (**Figure** **7**a‐i) can detect the temperature from the much longer recovery time (negligible recovery time induced by the pressure, Figure 7a‐ii).^[^ ^89^ ^]^ After determining the temperature from the independent feature, the pressure can be obtained from the resistance change at the given temperature (Figure 7a‐iii,iv). The PVDF arranged with the forward polarization can decouple pressure and temperature with improved precision, as two voltage signals used for decoupling are opposite in the sign (Figure 7b).^[^ ^90^ ^]^ The opposite sign in the voltage signals results from the opposite flow of electrons: increased polarization density from the applied pressure drives electrons to flow from the ground to the electrode, whereas decreased polarization density from elevated temperature forces electrons to flow from the electrode to the ground due to reduced electric dipole moments and expanded volume (Figure 7b‐i). With a positive‐negative bimodal peak in the voltage–time response curve, *V*_1_ results from both temperature and pressure, whereas *V*_2_ only comes from the temperature (Figures 7b‐ii,iii). Therefore, pyroelectric voltage (*V*_2_) that is linearly proportional to the temperature increase (in the range of temperature response time) gives the temperature. The pressure can then be obtained from the pressure‐dependent voltage [*V*_P_ = *V*_1_ − (*t*_1_/*t*_2_)*V*_2_], where *t*_1_ and *t*_2_ are the response times of *V*_1_ and *V*_2_, respectively. PVDF can also be combined with resistive materials to decouple pressure and temperature due to the different response behaviors in the two materials. For instance, the PVDF and rGO composite with an interlocked structure in the E‐skin exhibits a relatively instantaneous pyroelectric response to temperature and static piezoelectric response to pressure (Figure 7c).^[^ ^91^ ^]^ As the temperature does not affect the equilibrium resistance due to the relatively instantaneous response, the pressure can be directly measured from the equilibrium resistance. The temperature can then be detected by taking the difference between instantaneous and equilibrium resistance response, as demonstrated in the example with loading and unloading cycles of simultaneously applied pressure and temperature (Figure 7c‐ii).

It is also possible to explore materials with a much longer response time to temperature than that to pressure for decoupling input signals^[^ ^92^, ^93^, ^95^ ^]^ On one hand, force‐dependent structures such as porous or interlock structures are often used to achieve rapid response to pressure due to quickly changed conductive pathways; on the other hand, the material with a much longer response time to temperature is preferred. For example, an interlocked quasi‐hemispherical micro‐patterned E‐skin based on single‐walled CNTs (SWCNTs) and thermoplastic polyurethane (TPU) (Figure 7d‐i) rapidly responds to pressure (Figure 7d‐ii) and slowly responds to temperature.^[^ ^92^ ^]^ The enhanced conductivity of SWCNTs from thermal activation gives a response/recovery time that is nearly 1000 times of that to pressure, leading to the determination of the pressure from the rapid change of the resistance. The further changes after the inflection point (Δ*t* in the figure) provide the information to calculate the temperature (Figure 7d‐iii).

Hydrogels with a much longer response time to temperature changes than pressure,^[^ ^93^, ^95^ ^]^ also have the potential to decouple the two. In the example with an ionic conductive hydrogel consisting of poly(*N*‐isopropylacrylamide)‐acrylamide) (PNIAAm‐AAm), poly(vinyl alcohol) (PVA)‐GO and polyacrylic acid‐Fe^3+^ (PAA‐Fe^3+^) (Figure 7e),^[^ ^93^ ^]^ the relatively rigid PNIAAm‐AAm network provides the sensor with a quick response (<0.5 s) to pressure from decreased resistance (Figure 7e‐i,ii). In contrast, the temperature‐induced movement of free ions and volume changes of the polymer (Figure 7e‐iii) are associated with a long response time (>50 s, Figure 7e‐iv). Consequently, the slow structure change from the temperature allows the hydrogel sensor to successfully decouple the temperature and pressure (Figure 7e‐v).

## 7 Combined Structure and Material Innovations in a Single Sensing Unit

**Figure 8:**
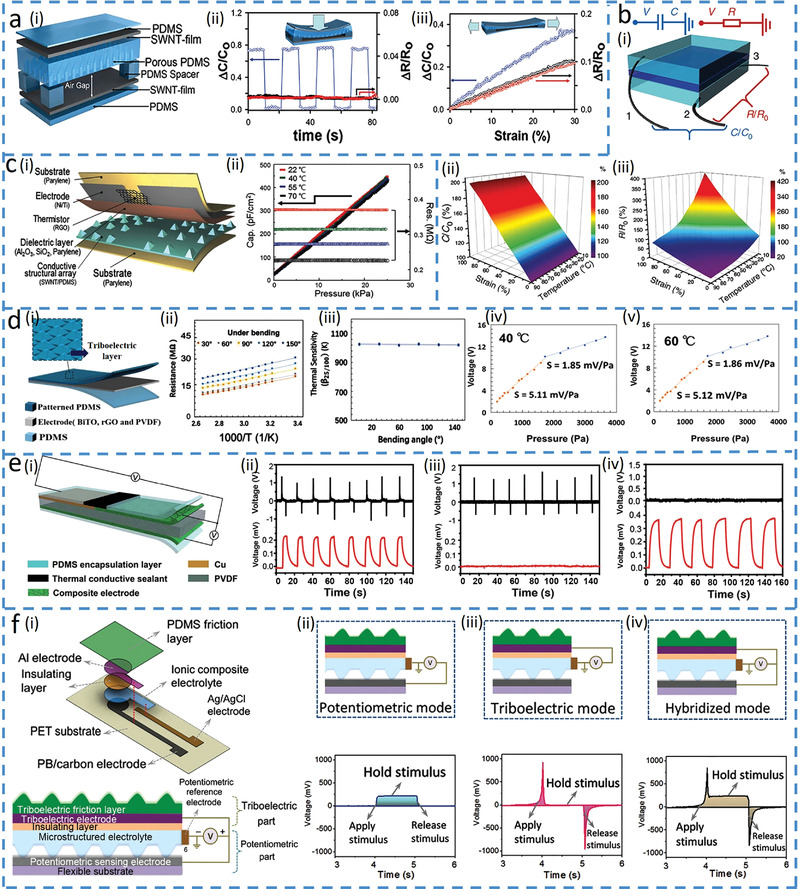
Combined structure and material innovations in a single sensing unit. a) Stretchable tactile sensor to distinguish multiple mechanical stimuli. Reproduced with permission.^[^ ^96^ ^]^ Copyright 2014 Wiley‐VCH. i) Schematic of the sensor and its responses in capacitance and resistance upon ii) pressure and iii) strain loading. b) Supramolecular E‐skin to decouple strain and temperature. Reproduced under the terms of the Creative Commons CC BY License.^[^ ^97^ ^]^ Copyright 2018, The Authors, published by Nature Publishing Group. i) Schematic of the sensor and its responses in capacitance and resistance. ii) Capacitance–strain curve of the sensor at different temperatures. iii) Resistance changes of the sensor upon varying strain and temperature. c) Linear bimodal pressure and temperature sensor^[^ ^98^ ^]^ with its i) schematic diagram and ii) simultaneous measurement of temperature and pressure. Reproduced with permission.^[^ ^98^ ^]^ Copyright 2018 Wiley‐VCH. d) Tactile E‐skin based on triboelectric and thermoresistive effects. Reproduced with permission.^[^ ^99^ ^]^ Copyright 2020 Elsevier Ltd. i) Schematic of the tactile sensor. ii) Relationship between the electrode resistance and temperature under different bending angles. iii) Thermal sensitivities of the sensor under different bending angles. Output voltage versus the applied pressure at iv) 40 and v) 60 °C, showing the temperature insensitivity. e) Dual‐parameter sensor with thermoelectric and piezoelectric sensing mechanisms. Reproduced with permission.^[^ ^100^ ^]^ Copyright 2019, The Royal Society of Chemistry. i) Schematic illustration of the thermoelectric and piezoelectric testing principles. Thermoelectric (red) and piezoelectric (black) responses of the sensor under ii) finger touch, iii) marker pen touch, and iv) non‐contact heating conditions. f) Mechanoreceptor based on a hybrid potentiometric‐triboelectric measuring principle. Reproduced with permission.^[^ ^101^ ^]^ Copyright 2020, Wiley‐VCH. i) Schematic of the mechanoreceptor and its ii) potentiometric, iii) triboelectric, and iv) hybrid modes.

The novel sensing materials can also be combined with the innovative structures to realize multimodal sensing in a single unit. As a simple example, the electrode in parallel‐plate capacitors with piezoresistive or thermal resistive properties can provide the sensor with an additional sensing modality, such as the piezoresistive electrode to decouple strain and pressure^[^ ^96^, ^102^, ^103^ ^]^ While the pressure decreases the gap and increases the capacitance of the tactile capacitor consisting of two SWCNT electrodes and a porous PDMS (**Figure** **8**a‐i,ii),^[^ ^96^ ^]^ the tensile strain stretches the SWCNT electrode to reduce the gap due to Poisson's effect for varying both resistance and capacitance (Figure 8a‐iii). The simultaneously applied pressure and tensile strain can be decoupled, as the resistance change from the electrode directly gives the tensile strain and the pressure is then derived from the capacitance at a given tensile strain.

Exploiting the thermal resistive stretchable electrode in the capacitive sensor can decouple the temperature and strain,^[^ ^97^, ^104^ ^]^ For example, adding conductive NaCl solution in hydrogels can provide stretchable electrodes in the capacitor to detect pressure‐induced strain due to reduced gap and increased capacitance and temperature‐induced ionic resistance changes (Figure 8b‐i).^[^ ^97^ ^]^ The capacitance change is insensitive to temperature variations (Figure 8b‐ii), leading to the direct determination of the strain. While the ionic resistance of the hydrogel decreases with increasing temperature as ionic liquids^[^ ^105^ ^]^ and many electronic conductors,^[^ ^106^ ^]^ the resistance also increases with the strain due to strain‐induced expansion of electrodes. The tensile strain determined from the capacitance can then be used to give the temperature from the resistance measurement (Figure 8b‐iii). The other mechanical deformations may also change the shape of the thermal resistor due to its flexibility and stretchability, so there is a benefit to separating the graphene‐based thermal sensing unit (rGO layer) in space to avoid its deformation by mechanical stimuli (Figure 8c‐i).^[^ ^98^ ^]^ The rGO layer bridges between two coplanar top electrodes and its projection on the bottom electrode does not have a raised pyramid to avoid the stress on the rGO under pressure. For the simultaneously applied pressure and temperature, the capacitor detects the pressure and the resistor measures the temperature with no interference between the two (Figure 8c‐ii). More importantly, orthogonal capacitive reactance and resistance in the phasor calculation of the impedance allow the calculation of the pressure and temperature from the imaginary and real components of the impedance, respectively.

Considering the fact that mechanical stimuli often affect both capacitor and resistor to cause cross‐sensitivity in the output signals, combining thermal‐sensitive electrodes with piezoelectric/ triboelectric materials can be advantageous to decouple temperature and pressure without cross‐sensitivity,^[^ ^99^, ^100^ ^]^ For example, the E‐skin is easily formed by placing a triboelectric top PDMS layer with pyramidal microstructures over a bottom thermosensitive bismuth titanate (BiTO) and rGO electrode layer (Figure 8d‐i).^[^ ^99^ ^]^ The deformed microstructure on the PDMS upon pressing induces triboelectric voltage output with practically no effect on the resistance electrode (Figure 8d‐ii,iii), whereas temperature merely changes electrode resistance without affecting voltage (Figure 8d‐iv,v). Combining the thermoelectric material as the electrodes for temperature sensing with piezoelectric material for pressure sensing can also decouple the two (Figure 8e).^[^ ^100^ ^]^ The piezoelectric PVDF as the middle layer responsible for pressure detection is sputter coated with Cu on the left and solution coated with hybrid polyaniline (PANI) on the right (Figure 8e‐i). The thermoelectric couple formed by the Cu and PANI‐based composite generates Seebeck voltage with increased temperature to detect temperature. Therefore, the thermoelectric and piezoelectric layers are independent without interference (Figure 8e, ii–iv).

Shared electrodes in a multimodal sensing unit can also provide a small footprint and a representative example is highlighted as a mechanoreceptor that combines the potentiometric and triboelectric sensing layer in a single cell to detect both static and dynamic stimuli without cross‐sensitivity (Figure 8f).^[^ ^101^ ^]^ The potentiometric part consists of microstructured ionic composite (PVA/NaCl/glycerol) with sensing (Prussian blue modified graphite carbon) and reference electrodes (Ag/AgCl), which is placed beneath the triboelectric part (patterned PDMS) as shown in Figure 8f‐i. The potentiometric part responds to static or slowly varying stimuli due to the increased contact area (Figure 8f‐ii), whereas the triboelectric sensing mode only creates momentary spikes at the start and end of stimulations (unable to sustain signal outputs for the holding stimuli) (Figure 8f‐iii). In addition, a hybrid signal output is generated when a stimulus with medium speed is applied, as both sensing mechanisms dominate (Figure 8f‐iv).

## 8 Conclusions and Potential Perspectives

The functional characteristics of human skins have inspired the rapid development of the flexible electronic devices with innovations in material selections, structural architectures, sensing mechanisms, and signal processing. The review briefly summarizes the recent advances in novel material, structures, sensing mechanisms, and their combinations for creating the truly multimodal sensors to decouple different input physical and mechanical stimuli. The representative methods to eliminate the cross‐sensitivity include the use of suppression of the interference, integrated multi‐sensing platforms, single sensing units with multiple sensing mechanisms, materials with different response times to different input stimuli, and combined structure and material innovations. The relationship among the materials’ properties, structure designs and the sensing mechanisms to decouple different stimuli provides multimodal sensors with more accurate measurements, while opening up the potential applications in more complex environments. Numerous applications have benefited from multimodal sensors with decoupled sensing mechanisms, including robotics, wearable health‐monitoring devices and human–machine interfaces. For instance, the robotic hand may obtain more accurate tactile information about the target object based on the discussed decoupling methods,^[^ ^101^, ^107^, ^108^ ^]^ for dexterous object manipulation. Wearable electronics can also detect decoupled biophysical signals (e.g., body temperature and movement with the artificial multimodal receptor^[^ ^12^ ^]^) for accurate real‐time health monitoring. Moreover, stretching/bending‐insensitive sensors avoid deformation‐induced performance degradation in flexible displays and other devices used in the human–machine interface,^[^ ^37^, ^109^ ^]^ with opportunities in metaverse augmented reality and virtual reality. Although significant progress has been made, the field of truly multimodal sensors is still in its infancy, with enormous challenges and opportunities for future developments.

1. Although using the same type of electrical signals^[^ ^90^, ^91^, ^92^, ^93^ ^]^ can decouple different input stimuli with distinct signal responses (e.g., magnitude and/or response/recovery times), it is often accompanied by the degraded sensing performance of the multimodal sensors (e.g., longer sensing time,^[^ ^91^, ^93^ ^]^ or lower sensitivity^[^ ^92^ ^]^). As a result, combining different sensing mechanisms or even the types of electrical signals (e.g., voltage and resistance) to decouple varying input stimuli is more promising.
2. While many strategies have been developed to decouple two input signals, the measurement of three different physical stimuli with a single sensing unit is rarely reported. One solution is to create more sensing properties/mechanisms in the active materials (e.g., hydrogel nanocomposites^[^ ^110^ ^]^ with excellent biocompatibility). Inspirations can be drawn from the example, where PEDOT:PSS with combined electronic and ionic conductivity provides the decoupling method for temperature, pressure and humidity.^[^ ^77^ ^]^ As the coupling of three input signals in the output is challenging, the development and use of the sensing mechanism with an exclusive response to one input can help reduce the complexity, where the inspirations can draw from the deformation‐insensitive sensors. For example, it could be of interest to integrate multiple sensors with suppressed interference or reference sensors sensitive to only one stimulus into a multi‐sensing platform for decoupled multimodality.
3. The mass production of the multimodal sensor for applications in robots or prostheses can be challenging for the multi‐sensing platforms that explore different sensors with various mechanisms, because of the complex fabrication process and high materials cost. To scale up the device fabrication out of the laboratory, it is desirable to employ sensors with additional sensing mechanisms (provided by electrodes),^[^ ^97^, ^99^, ^100^ ^]^ or shared electrodes^[^ ^101^ ^]^ in different sensing layers. Besides the optimized configuration, it is of high interest to explore low‐cost manufacturing methods for fabricating multifunctional material composites in the multimodal sensors.^[^ ^111^, ^112^, ^113^ ^]^

## Conflict of Interest

The authors declare no conflict of interest.
